# Outcomes and Complications of Minimally Invasive Surgery of the Lumbar Spine in the Elderly

**DOI:** 10.7759/cureus.519

**Published:** 2016-03-05

**Authors:** Mauricio J Avila, Christina M Walter, Ali A Baaj

**Affiliations:** 1 Neurological Surgery, NewYork-Presbyterian/Weill Cornell Medical College; 2 Neurological Surgery, The University of Arizona

**Keywords:** minimally invasive surgery, aged, patient outcome assessment, spine, adult

## Abstract

Introduction: Minimally invasive spine (MIS) surgery is gaining popularity in the elderly. With aging population and a strong desire for all patients to remain physically active, this trend will likely continue. Previous studies have reported clinical outcomes in the elderly undergoing MIS surgery; however, most of these studies encompass multiple surgeons at different sites and thus present heterogeneous experiences. In this work, we investigate the clinical outcomes and complications of all lumbar MIS procedures performed in patients over 65 years of age by a single surgeon.

Methods: A retrospective analysis of a prospectively maintained database of spine surgeries was performed. Twenty-six patients who underwent 27 procedures were included.

Results: Mean age at surgery was 72 years (range 64-86). The mean BMI was 30.2 kg/m^2^, patients had an average of 5 comorbidities, took 9 medications, and 15% were smokers. The mean symptoms duration was 40.6 months with the numeric rating scale (NRS) and the Oswestry disability index (ODI) prior to surgery of 7.68 and 50% respectively. Six different types of procedures were performed, the most common was the interlaminar decompression and fusion (ILIF) followed by MIS laminectomy, microdiscectomy and MIS lateral fusion (XLIF). 74% of the surgeries were done at a single level. Average blood loss was 43 mL, and the mean length of stay was 1.7 days. There were three complications (11.1%): one urinary tract infection, one pulmonary embolism, and one new, postoperative weakness. At six months follow-up, there was a mean improvement of 27% in ODI, and a 5.6 improvement in NRS (both p<0.05); 90% of patients stated they would have the surgery again.

Conclusion: Minimally invasive lumbar spine surgery is both safe and highly effective in the elderly population. Patient selection is of utmost importance. This data will add to the existing literature on the overall safety and effectiveness of these procedures in the elderly population.

## Introduction

The fastest growing segment of the population in the United States are individuals over 65 years of age [1-2], with projections of a further increment of 16.5% by 2020 [1]. The increasing number of older adults worldwide represents a challenge to any healthcare system [3].

Epidemiological data shows that back pain is the most common musculoskeletal condition affecting any population [4-5] with a prevalence of 4-33% at any given time [4].This number is even higher in older adults with an estimated prevalence of 56% [6]. The high prevalence and the disability of a chronic condition such as back pain can limit the normal functioning of an older adult compared to those without back pain [7-8]. As older adults are living longer free of disabilities [3], it is important to find a way to safely treat older patients with low back pain.

Geriatric patients represent a surgical challenge due to their comorbidities and normal physiological changes with aging [2, 9]. Minimally invasive spine (MIS) surgery is aimed at avoiding complications related to the open procedures with less disruption of the normal anatomy, less blood loss and shorter hospitalizations [10-13]. Previous research has shown the feasibility of MIS surgery in older patients [14-15]; however, the data was retrieved from different surgeons in one or multiple centers.

The aim of this study was to evaluate outcomes and complications of patients over 65 treated with MIS surgery of the lumbar spine by a single surgeon in a single institution.

This work was approved by the Human Subjects Protection Program-University of Arizona.

## Materials and methods

A retrospective chart review from a prospectively maintained database was performed until December 2014 for patients over 65 years of age who underwent minimally invasive lumbar spine surgery by a single surgeon in a single center. Our inclusion criteria were: patients who were 65 years or older at the time of surgery, patients who had any lumbar degenerative spine pathology, patients who had a minimally invasive procedure, and patients who had appropriate clinical and radiographic follow-up.

Patients’ demographics (age, weight, comorbidities, medications etc.), length of stay, estimated blood loss, immediate complications, surgical morbidity, mortality, and type of procedure were retrieved. The patients' reported outcomes such as numerical rating scale (NRS) for pain, and Oswestry disability index (ODI) were retrieved before surgery and at every follow-up (starting at week six) after surgery. We used paired sample t-test to compare means between groups and set a statistical significance of p<0.05. We also used measures of central tendency for continuous variables. Data was retrieved and processed with Microsoft Excel. Statistical analysis was performed with SPSS software (IBM) version 20.

## Results

There was a slight predominance of female patients in this series (54%). The mean age at surgery was 72 years old with a range of 65 to 86 years. Overall, the mean BMI was 30.2 kg/m^2^ (Obesity). The mean duration of patients’ symptoms before surgery was 40 months, and 10% of the patients had a previous spine surgery. The mean estimated blood loss (EBL) during surgery was 43.9 mL, and the mean length of stay was 1.6 days. The most common affected level was L4-L5. The complete patients’ demographics are presented in Table 1.

**Table 1:** Baseline patients' characteristics

Characteristic	Value
Age (range)	72 (65-86)
Sex	
Male (%)	12 (46)
Female (%)	14 (54)
BMI (range)	30.2 (17.4-39)
Comorbidities (range)	5 (2-10)
Medications(ranges)	9 (3-24)
Smoker (%)	4 (15.4)
Symptoms duration in months (range)	40.6 months (0.5-180)
Number of previous non-surgical treatment (range)	2.5 (1-4)
Previous spine surgery (%)	10 (38.5)
Estimated blood loss (range)	43.85 mL (10-300)
Length of stay (range)	1.65 days (1-5)
Follow up (range)	7.8 months (2-24)
Affected level	
L1-L2 (%)	1 (3.7)
L2-L3 (%)	2 (7.4)
L3-L4 (%)	3 (11.1)
L4-L5 (%)	11 (40.7)
L5-S1 (%)	3 (11.1)
Multiple levels (%)	7 (26)

**Figure 1:**
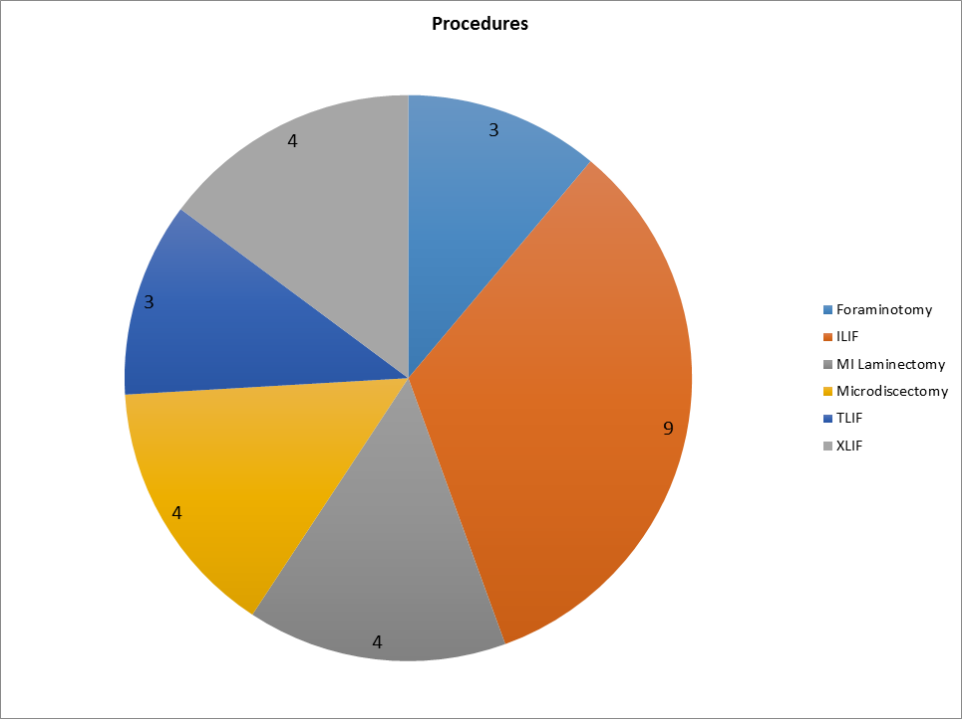
Type of MIS procedures performed ILIF: Interlaminar Lumbar Interbody Fusion TLIF: Transforaminal Interbody Fusion XLIF: Extreme Lateral Interbody Fusion MI: Minimally Invasive

Six different types of procedures were performed (Figure 1); the most common was the interlaminar decompression and fusion (ILIF) followed by MIS laminectomy, microdiscectomy, and MIS lateral fusion (XLIF).

Regarding patients reported outcomes (Table 2), the greater difference for NRS score was seen at three months with a mean difference of 7 points compared to baseline. Moreover, the difference in NRS was statistically significant at all the different follow-ups (six weeks, three months, six months, and at one year) after surgery.

For the ODI, the greater difference (37%) was also seen at three months. The results for ODI were also statistically significant at all the follow-up dates after the procedure. After six months there was an overall improvement of 27% for ODI compared to baseline ODI (p=0.018). 90% of the patients answered that they will have the surgery again.

**Table 2:** Results of the patients’ reported outcomes

Comparison		Mean Difference of the Pair	95% Confidence Interval	p Value	
NRS PreOp	NRS 6 weeks	4.95	2.67-7.23	0.001	
	NRS 3 months	7.0	0.72-5.44	<0.001	
	NRS 6 months	5.63	4.22-7.04	<0.001	
	NRS 1 year	4.94	2.91-6.97	0.001	
ODI PreOp*	ODI 6 weeks	0.28	0.05-0.51	0.029	
	ODI 3 months	0.37	0.24-0.51	0.003	
	ODI 6 months	0.27	0.007-0.47	0.018	

One patient had an NRS and ODI score at two years of follow-up of two and 18% respectively.

There were three complications in this study (11.1%). One patient had a urinary tract infection, one patient had a pulmonary embolism diagnosed after surgery that did not carry any clinical consequences for the patient, and finally one patient had right hip flexors and knee extensor weaknesses noticeable at one month after surgery; however, at six months, the patient improved to near normal strength after physical therapy and rehabilitation.

There was no mortality in this cohort.

## Discussion

With the global population aging and life expectancy surpassing 80 years, it is expected that spine surgeons will encounter more elderly patients in their practice [1].

This study shows the feasibility of minimally invasive spine procedure in patients older than 65 years of age performed by a single surgeon in one center. Although aimed at over 65 years, our sample had a mean age of 72 years with one patient who safely underwent surgery at 86 years.

There is a growing body of evidence showing the clinical benefits of treating patients over 65 years old with surgery [14-16]. For this study, we did not include patients who had traumatic fractures; however, it is important to recognize that fractures in the elderly population have increased in frequency over the last few years [17]. The indications for surgery in these patients may be different than for those suffering from degenerative changes [17-18] as well as the profile of complications [17].

Our results show a statistically significant difference (improvement) of the NRS and ODI score starting at two weeks after the surgery. More importantly, is that the results for the ODI score were all above a 10% difference, which is required to be considered a “clinically significant” difference [10, 19]. Particularly, ODI improved 28% at six weeks after surgery, 37% at three months after surgery, and 27% at six months after surgery.

Our complications were transient and all resolved at the patients’ last follow-up without any residual injuries. The latter is in accordance of what has been reported in the literature [14-15]. Despite that 38% of the patients had a previous spine surgery, this fact did not alter our complications rate as only one patient (urinary tract infection) of the three patients with complications had a previous spine surgery; in fact this complication was not directly related to the lumbar spine pathology or the previous surgery.

When approaching a surgery for an elderly patient, it is crucial to have a team of health professionals who allows the patient to be in the best physiological state as possible before surgery [2, 9]. Figure 1 shows the different type of procedures done in this case series. Several MIS are represented and, aside for 9 ILIF, there was not any clear dominance of one procedure over the others which show how versatile MIS can be for elderly patients. Minimally invasive approaches allow treating lumbar spine pathologies with less disruption of the anatomy and less blood loss compared with open procedures. In cases of patient with multiple comorbidities, it is more desirable to perform MIS surgery due to their mentioned benefits. Furthermore, if the spine surgeon is comfortable with several MIS procedures, the range of elderly patients who could benefit from surgery would increase.

Patient selection before surgery is a key step to succeed when approaching elderly patients. It is of utmost importance to determine the patient’s pre-surgical, usual functioning status and have clear goals for the surgery with the patient and family. The evaluation of comorbidities and family support are necessary to ensure a successful procedure. In our cohort, patients had a mean of five comorbidities and were taking nine medications at the time of surgery, which could explain some of the complications [20]. However, as not all the patients with comorbidities had complications, this also shows that having comorbidities does not necessarily preclude surgery in the elderly population.

This study has some limitations. First, it is a retrospective study; nonetheless, we collected the data in a prospective fashion as part of an ongoing database for spinal procedures. Second, some patients did not complete the full scoring system of the ODI, which could have shown more pronounced differences; however, we believe that despite this, the trend is clear toward a clinical and statistically significant difference. Finally, the relatively small sample of this study may limit the generalizability of our results. Nonetheless, as shown previously by other studies [14-15], there are clear benefits in doing surgery in elderly patients.

## Conclusions

Minimally invasive spine surgery in patients over 65 years of age with lumbar degenerative spine pathologies is safe and yields excellent outcomes. Newer surgical techniques allow spine surgeons to offer solutions to high-risk patients with lumbar spine pathologies who wouldn’t otherwise tolerate traditional open-spine procedures.
